# *SCN5A* variant type-dependent risk prediction in Brugada syndrome

**DOI:** 10.1093/europace/euaf024

**Published:** 2025-02-11

**Authors:** Takanori Aizawa, Takeru Makiyama, Hai Huang, Tomohiko Imamura, Megumi Fukuyama, Keiko Sonoda, Koichi Kato, Takashi Hisamatsu, Yuko Nakamura, Kenji Hoshino, Junichi Ozawa, Hiroshi Suzuki, Kazushi Yasuda, Hisaaki Aoki, Takashi Kurita, Yoko Yoshida, Tsugutoshi Suzuki, Yoshihide Nakamura, Yoshiharu Ogawa, Shintaro Yamagami, Hiroshi Morita, Shinsuke Yuasa, Masakazu Fukuda, Makoto Ono, Hidekazu Kondo, Naohiko Takahashi, Seiko Ohno, Yoshihisa Nakagawa, Koh Ono, Minoru Horie

**Affiliations:** Department of Cardiovascular Medicine, Kyoto University Graduate School of Medicine, 54 Shogoin Kawahara-cho, Sakyo-ku, Kyoto 606-8507, Japan; Department of Cardiovascular Medicine, Kyoto University Graduate School of Medicine, 54 Shogoin Kawahara-cho, Sakyo-ku, Kyoto 606-8507, Japan; Department of Cardiovascular Medicine, Kyoto University Graduate School of Medicine, 54 Shogoin Kawahara-cho, Sakyo-ku, Kyoto 606-8507, Japan; Department of Cardiovascular Medicine, Kyoto University Graduate School of Medicine, 54 Shogoin Kawahara-cho, Sakyo-ku, Kyoto 606-8507, Japan; Department of Cardiovascular Medicine, Shiga University of Medical Science, Otsu, Japan; Medical Genome Center, National Cerebral and Cardiovascular Center, Suita, Japan; Department of Cardiovascular Medicine, Shiga University of Medical Science, Otsu, Japan; Department of Public Health, Dentistry and Pharmaceutical Science, Okayama University Graduate School of Medicine, Okayama, Japan; Department of Pediatrics, Tsuchiura Kyodo General Hospital, Tsuchiura, Japan; Department of Cardiology, Saitama Children’s Medical Center, Saitama, Japan; Department of Pediatrics, Niigata University Graduate School of Medical and Dental Sciences, Niigata, Japan; Uonuma Institute of Community Medicine, Niigata University Medical and Dental Hospital, Niigata, Japan; Department of Pediatric Cardiology, Aichi Children’s Health and Medical Center, Obu, Japan; Department of Pediatric Cardiology, Osaka Women’s and Children’s Hospital, Izumi, Japan; Division of Cardiovascular Center, Kindai University School of Medicine, Osakasayama, Japan; Division of Pediatric Cardiology and Electrophysiology, Osaka City General Hospital, Osaka, Japan; Division of Pediatric Cardiology and Electrophysiology, Osaka City General Hospital, Osaka, Japan; Division of Pediatric Cardiology and Electrophysiology, Osaka City General Hospital, Osaka, Japan; Division of Cardiology, Hyogo Prefectural Kobe Children’s Hospital, Kobe, Japan; Department of Cardiology, Tenri Hospital, Tenri, Japan; Department of Cardiovascular Therapeutics, Faculty of Medicine, Dentistry and Pharmaceutical Sciences, Okayama University, Okayama, Japan; Department of Cardiovascular Medicine, Faculty of Medicine, Dentistry and Pharmaceutical Sciences, Okayama, Japan; Division of Cardiology, Department of Medicine and Clinical Science, Yamaguchi University Graduate School of Medicine, Ube, Japan; Division of Cardiology, Department of Medicine and Clinical Science, Yamaguchi University Graduate School of Medicine, Ube, Japan; Department of Cardiology and Clinical Examination, Faculty of Medicine, Oita University, Yufu, Japan; Department of Cardiology and Clinical Examination, Faculty of Medicine, Oita University, Yufu, Japan; Medical Genome Center, National Cerebral and Cardiovascular Center, Suita, Japan; Department of Cardiovascular Medicine, Shiga University of Medical Science, Otsu, Japan; Department of Cardiovascular Medicine, Kyoto University Graduate School of Medicine, 54 Shogoin Kawahara-cho, Sakyo-ku, Kyoto 606-8507, Japan; Department of Cardiovascular Medicine, Shiga University of Medical Science, Otsu, Japan

**Keywords:** Brugada syndrome, *SCN5A*, Lethal arrhythmia event, Variant type, Loss of function

## Abstract

**Aims:**

The variant in *SCN5A* with the loss of function (LOF) effect in the cardiac Na^+^ channel (Na_v_1.5) is the definitive cause for Brugada syndrome (BrS), and the functional analysis data revealed that LOF variants are associated with poor prognosis. However, which variant types (e.g. missense or non-missense) affect the prognoses of those variant carriers remain unelucidated.

**Methods and results:**

We defined *SCN5A* LOF variants as all non-missense and missense variants that produce peak *I*_Na_ < 65% of wild-type previously confirmed by patch-clamp studies. The study population consisted of 76 Japanese BrS patients (74% patients were male and the median age [IQR] at diagnosis was 28 [14–45] years) with LOF type of *SCN5A* variants: 40 with missense and 36 with non-missense variants. Non-missense variant carriers presented significantly more severe cardiac conduction disorder compared to the missense variant carriers. During follow-up periods of 9.0 [5.0–14.0] years, compared to missense variants, non-missense variants were significant risk factors of lifetime lethal arrhythmia events (LAEs) (*P* = 0.023). When focusing only on the missense variants that produce no peak *I*_Na_, these missense variant carriers exhibited the same clinical outcomes as those with non-missense (log-rank *P* = 0.325). After diagnosis, however, both variant types were comparable in risk of LAEs (*P* = 0.155).

**Conclusion:**

We identified, for the first time, that *SCN5A* non-missense variants were associated with higher probability of LAE than missense variants in BrS patients though it did not change significantly after diagnosis.

## Graphical Abstract

**Graphical Abstract:**
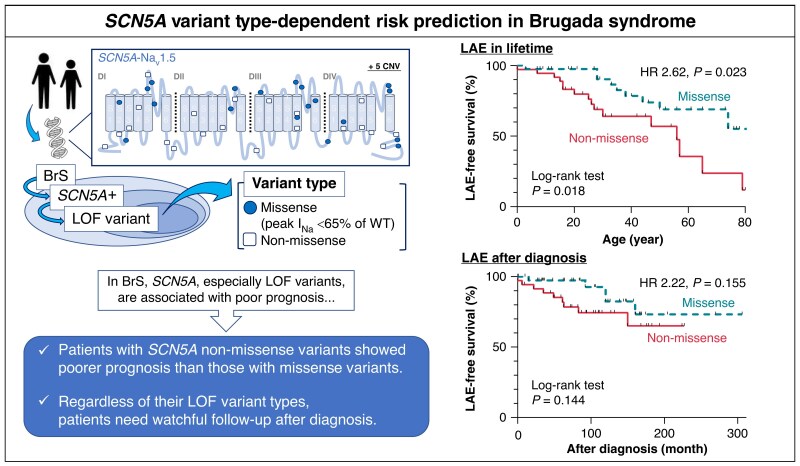
BrS, Brugada syndrome; CNV, copy number variation; D, domain; HR, hazard ratio; LAE, lethal arrhythmia event; LOF, loss of function; S, segment; WT, wild-type.

**What’s new?**

- The loss of function (LOF) variants of *SCN5A* are associated with Brugada syndrome (BrS), and, moreover, functionally validated LOF variants linked to poor prognosis. However, it remains unelucidated which types of variants (e.g. missense or non-missense) affect clinical outcomes.
- Our study revealed that BrS patients with non-missense variants showed not only more severe conduction disorder but higher probability of arrhythmia outcomes through lifetime than those with missense variants. However, after patients were diagnosed with BrS, patients required adequate follow-up regardless of the variant types.
- Further analysis revealed that complete LOF missense variants (peak *I*_Na_ = 0) carriers exhibited comparable outcomes to non-missense carriers, indicating the sodium currents produced by each variant may have important factors for prognosis.

## Introduction

Variants of the *SCN5A* gene that encodes α-subunit of the cardiac sodium channel (Na_v_1.5) cause various genetic arrhythmias known as ‘sodium channelopathy’^1^ Particularly, *SCN5A* loss of function (LOF) are associated with arrhythmia disease including Brugada syndrome (BrS), idiopathic ventricular fibrillation, cardiac conduction defects, supraventricular tachycardia (SVT), or dilated cardiomyopathy.^2^

Since the first report of BrS,^3,4^ its genetic background has not yet been fully elucidated, but it is also true that accurate interpretation of the variants, especially *SCN5A*, has achieved some success.^5^ Though *SCN5A* variants could be detected in only 10–30% of BrS patients,^4,6^  *SCN5A* has been recognized as the causative gene for BrS^7^ resulting in sudden cardiac death and associated with poor prognosis.^8,9^ Furthermore, Ishikawa *et al*.^10^ reported that the functional reclassification of *SCN5A* variants helps predicting the prognosis of BrS patients effectively by setting the cut-off at 53.2–65.6% of wild-type (WT) for peak *I*_Na_.

*SCN5A* variants can be classified in two groups, missense variants and non-missense variants. Some of the missense variants cause trafficking defects, abnormal gating, or altered channel ion selectivity.^11^ Most of the non-missense variants, such as truncation, frameshift by insertion or deletion, and copy number variants, can cause nonsense-mediated mRNA decay (NMD).^12,13^ As Na_v_1.5 is traditionally thought to function without multimerization, non-missense variants may cause more severe phenotypes compared to missense variants due to haploinsufficiency, at variance with the case of *KCNQ1* and *KCNH2* whose missense variants lead to severe phenotypes due to their dominant negative effects.^14,15^

Therefore, several studies reported that non-missense *SCN5A* variants are associated with more severe clinical phenotypes, such as syncope or cardiac conduction disorder, in BrS.^16–18^ However, incidences of lethal arrhythmia events (LAEs) among LOF-type variants remain unelucidated. In this study, we sought to investigate the prognostic impact on LAEs between two different types of *SCN5A* variants (i.e. missense vs. non-missense) in BrS patients.

## Methods

### Study population

This study included BrS patients who underwent genetic testing between 1998 and 2023 at Shiga University of Medical Science or Kyoto University Graduate School of Medicine, and the patients harbouring LOF-type *SCN5A* variants were selected.

### Declaration of Helsinki

We obtained written informed consent from all the patients and their guardians per the guidelines approved by the institutional review boards of Shiga University of Medical Science and Kyoto University Graduate School of Medicine. This study complies with the Declaration of Helsinki.

### Genetic testing

Genomic DNA was extracted from the peripheral lymphocytes of each patient. Denaturing high-performance liquid chromatography (WAVE system Model 3500; Transgenomic, NE, USA) (between 1996 and 2014 for *KCNQ1*, *KCNH2*, *SCN5A*, *KCNE1*, and *KCNE2*) was used for initial screening. From 2014, targeted gene panel sequencing using HaloPlex HS kit (Agilent Technology, CA, USA) including more than 45 genes and MiSeq system (Illumina, CA, USA) were used for genetic testing. All identified variants were verified by Sanger sequencing.^19^

In patients whose pathogenic variants of the targeted genes were not detected, we performed MLPA analysis using probes from the SALSA MLPA Kit P108 (MRC Holland, Amsterdam, The Netherlands), and the detected copy number variants (CNVs) were confirmed by Sanger methods as previously reported.^20^

The complementary DNA sequences of *SCN5A* were based on the GenBank reference sequence NM_198056.2.

### Interpretation and classification of *SCN5A* variants

To select the missense variants, *SCN5A* LOF variants were defined if their peak *I*_Na_ were <65% of that produced by wild-type (WT)^7^ and only LOF missense variants were included by reviewing past experimental studies (see Supplementary material online, *Table S1*). In addition, some variants were defined as complete loss of function (cLOF) when they did not produce any sodium currents (*I*_Na_), while the others were defined as partial LOF (pLOF) when they produced *I*_Na_ (peak *I*_Na_ between 0% and 65% of the WT *I*_Na_). Non-missense variants included nonsense, frameshift, in-frame deletion, canonical (± 2) splice site,^21^ and CNV^20^ that are expected to not produce *I*_Na_ (i.e. LOF). Their minor allele frequencies were checked using both the Genome Aggregation Database (gnomAD: https://gnomad.broadinstitute.org) and the National Bioscience Database Center’s integrated database of Japanese genomic variation (Togovar: https://grch37.togovar.org). Patients who harbour double *SCN5A* variants or other compound gene variants, which can cause inherited arrhythmia diseases including BrS, were excluded.

### Assessment of clinical characteristics and outcomes

Patients who showed type 1 Brugada pattern ST-elevations (i.e. coved type) were considered as BrS. Sinus node dysfunction (SND) was defined by slow heart rate (<50 bpm).^22^ SVT included atrial fibrillation, atrial tachycardia, or atrial flutter. Each ECG parameter was measured in lead II at diagnosis. QT was corrected for heart rate by the Bazzet’s formula (QTc).^23^ Fragmented QRS was defined as fragmentations in QRS complex: ≥4 spikes in one lead or ≥ 8 spikes in all of the V_1_–V_3_,^24^ and aVR sign was defined as R ≥ 0.3 mV or R/q ≥ 0.75 in lead aVR,^25^ and large S-wave in lead I was defined as the amplitude ≥ 0.1 mV or duration ≥ 40 ms.^26^ Early repolarization pattern was identified when J-point and ST segment elevation is > 1 mm in ≥ 2 contiguous leads.^27^ Shanghai score was calculated at diagnosis.^28^ Syncope was defined as unconscious states due to unknown cause or suspected cardiac arrhythmias. The endpoints of this study were LAEs including documented ventricular tachycardia or ventricular fibrillation, cardiopulmonary arrest, and sudden death with or without documentation of fatal arrhythmias.

### Statistics

Continuous variables were shown as the median (interquartile range) and categorical variables were shown as number (percentage). The differences in the clinical characteristics, ECG parameters, or follow-up data between the different LOF-type *SCN5A* variants were compared using the Mann–Whitney *U* test for continuous variables, and the χ^2^ or Fisher’s exact test for categorical variables. The probability of the first LAEs was estimated by the Kaplan–Meier method, and the differences were evaluated by the log-rank test. To predict the association between LAEs and predictor variables, the univariate Cox proportional hazard model was used. Criteria for censoring were defined as last follow-up or loss to follow-up. Two-tailed *P* of <0.05 was considered as statistically significant. All statistical analyses were performed by using JMP Pro version 17.0.0 (SAS Institute Inc.).

## Results

### Characteristics of the Brugada syndrome patients who harbour *SCN5A* variants

After the referral for genetic tests, we identified 76 patients who harboured LOF-type *SCN5A* variants from 58 unrelated families; 58 probands and 18 family members were included. The follow-up periods were 9.0 [5.0–14.0] years: our cohort contained 56 males (74%) and the median age at diagnosis was 28 [14–45] years old. There were 11 patients who were previously positive for pharmacological provocation tests and two of them showed spontaneous type 1 ECG during follow-up. LAEs occurred among 25 (33%) patients with an overall annual LAE rate of 881/100 000 person-year, and the average age at first events was 28 [16–49] years old. Five of the patients had LAEs during fevers.

**Figure 1:**
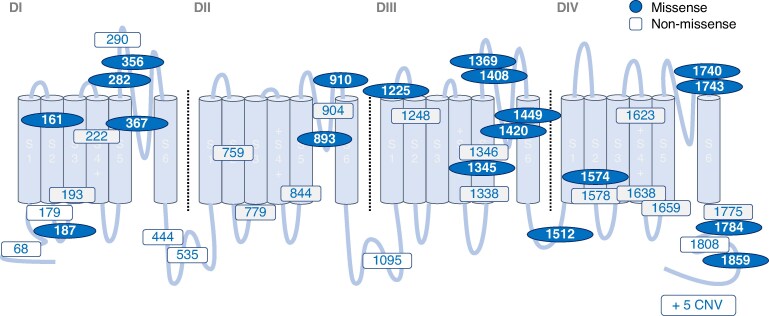
The location of *SCN5A* LOF variants within topology of the Nav1.5 alpha subunit. Location of variants identified in BrS patients. BrS, Brugada syndrome; CNV, copy number variation; D, domain; LOF, loss of function; S, segment.

*Figure 1* depicts the location of LOF variants detected on the *SCN5A* topology. The total number of variants was 48. Twenty-one variants were missense, and 14 of them were located in the pore region. Twenty-seven variants were non-missense variants, including 10 nonsense variants, 1 in-frame deletion, 10 frame shifts, 1 splicing site, and 5 CNVs (see Supplementary material online, *Table S1*).

### The clinical implication of the variant types: non-missense vs. missense *SCN5A*

We classified the participants into two groups depending on the variant types: 40 patients with missense variants and 36 with non-missense variants, and their clinical outcomes were compared. *Table 1* summarizes the characteristics of study subjects, and there were no significant differences between the two groups regarding the rate of family history of sudden death, Shanghai scores, and incidence of SND and SVT. Regarding the ECG parameters, the frequencies of spontaneous type 1 ECG were comparable between the two groups. However, compared to the missense variant carriers, the non-missense variant carriers presented significantly longer *P* wave duration (120 [111–128] vs. 110 [98–123], *P* = 0.008), longer PQ interval (212 [187–225] vs. 186 [152–212], *P* = 0.003), and wider QRS complex (120 [105–131] vs. 111 [96–123], *P* = 0.033).

**Table 1:** Clinical characteristics of BrS: missense vs. non-missense

	Missense (*n* = 40)	Non-missense (*n* = 36)	*P* value
Proband	29 (73)	29 (81)	0.410
Male	26 (65)	30 (83)	0.070
Age at diagnosis (years)	31 (8–48)	27 (16–42)	0.851
Family history of sudden death	17 (43)	12 (33)	0.411
Shanghai score	4.5 (4.0–6.0)	4.5 (4.1–7.0)	0.182
SND	11 (28)	16 (44)	0.123
Age at SND diagnosis (years)	28 (13–34)	25 (11–41)	0.540
SVT	10 (25)	7 (19)	0.562
Age at first SVT diagnosis (years)	43 (29–67)	29 (21–50)	0.230
LP positive	12/15 (80)	13/15 (87)	1.000
Inducible VT/VF at EPS	9/13 (69)	11/13 (85)	0.645
Spontaneous type 1 ECG	33 (83)	34 (94)	0.159
RR II (ms)	923 (751–1003)	901 (780–998)	1.000
P II	110 (98–123)	120 (111–128)	0.008
PQ II (ms)	186 (152–212)	212 (187–225)	0.003
QRS II (ms)	111 (96–123)	120 (105–131)	0.033
QTc (Bazett) II (ms)	403 (377–436)	416 (395–430)	0.281

Data are expressed as median (interquartile range) or number (%).

BrS, Brugada syndrome; ECG, electrocardiogram; EPS, electrophysiology study; LP, late potential; VF, ventricular fibrillation; VT, ventricular tachycardia; SND, sinus node dysfunction; SVT, supraventricular tachycardia.

### Non-missense variant carriers showed worse clinical outcomes through lifetime but not after diagnosis

**Figure 2:**
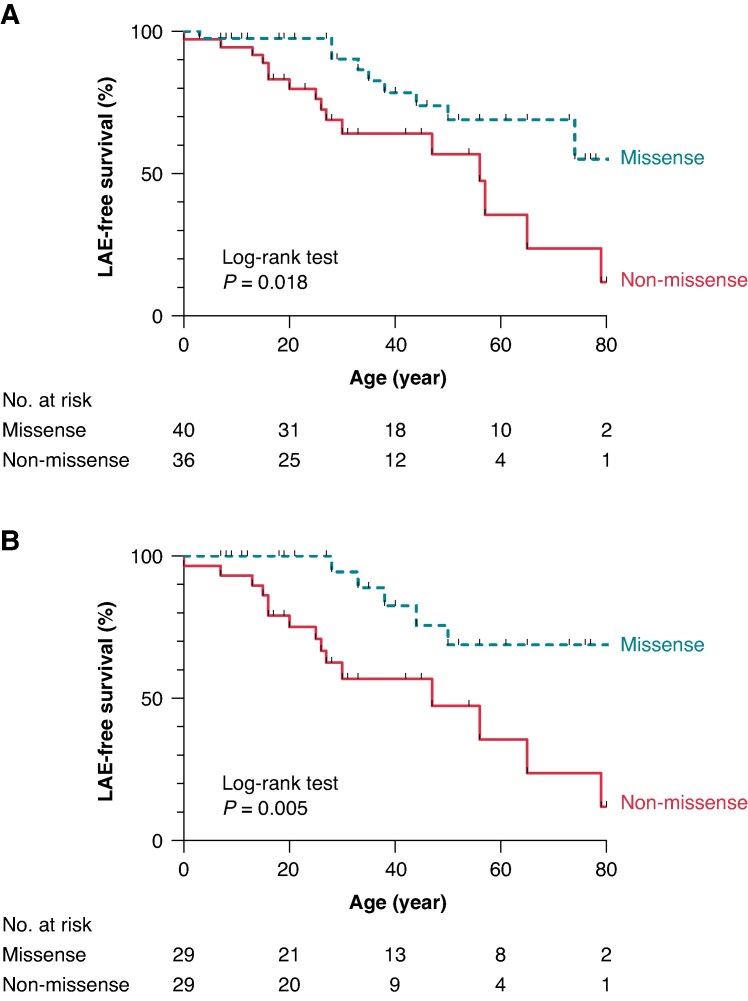
Kaplan–Meier survival analyses for the LAE-free survival. LAE-free survival estimates in lifetime for BrS patients with missense and non-missense variants. Overall patients (*A*) and probands only (*B*). BrS, Brugada syndrome; LAE, lethal arrhythmia event.

*Table 2* shows a comparison of the prognoses between the two groups (missense vs. non-missense). Although the incidence of cardiogenic syncope was comparable between the two groups, the incidence of LAEs was significantly higher in patients with non-missense variants than those with missense variants (44%; 1302/100 000 person-year vs. 23%; 573/100 000 person-year, *P* = 0.042). The Kaplan–Meier analysis shows that the probability of LAEs non-missense was significantly higher in variant carriers than in the carriers of missense variants (log-rank *P* = 0.018, *Figure 2*). When only the probands were analysed, this outcome became worse as shown in *Figure 2B* (log-rank *P* = 0.005). *Table 3* shows that non-missense variants were identified as a significant risk factor for lifetime LAEs by the univariate Cox regression analysis (hazard ratio [HR]: 2.62, 95% confidence interval [CI]: 1.15–5.98, *P* = 0.023).

**Table 2:** Follow-up data of BrS: missense vs. non-missense

	Missense (*n* = 40)	Non-missense (*n* = 36)	*P* value
Follow-up interval (years)	6.5 (3.0–13.0)	9.5 (6.0–14.8)	0.097
Syncope	10 (25)	11 (31)	0.589
Age at syncope (years)	33 (17–60)	17 (13–33)	0.240
LAE through lifetime	9 (23)	16 (44)	0.042
Per 100 000 person-years	573	1302	
Age at first LAE (years)	35 (28–47)	26 (15–54)	0.269
LAE after diagnosis	5 (13)	9 (25)	0.237
Per 100 000 person-years	1392	2426	
ICD implantation	15 (38)	19 (53)	0.181
2nd prevention	6 (15)	12 (33)	0.061
PM implantation	2 (5)	9 (25)	0.013
Device related complication	5/15 (33)	4/19 (21)	0.420
Drug therapy	11 (28)	6 (17)	0.258

Data are expressed as median (interquartile range) or number (%).

BrS, Brugada syndrome; ECG, electrocardiogram; ICD, implantable cardioverter defibrillator; LAE, lethal arrhythmia event; PM, pacemaker.

**Table 3:** Univariable Cox regression analysis for LAE

	Lifetime			After diagnosis		
Variable	HR	95% CI	*P* value	HR	95% CI	*P* value
Male	1.44	0.54–3.88	0.467	4.39	0.57–33.7	0.155
Family history of sudden death	0.61	0.25–1.46	0.264	0.89	0.30–2.65	0.830
SND	2.12	0.96–4.69	0.065	2.25	0.78–6.50	0.134
SVT	1.15	0.49–2.69	0.747	1.89	0.63–5.71	0.257
Spontaneous type 1 ECG	0.81	0.30–2.19	0.672	1.14	0.25–5.13	0.864
History of syncope	1.73	0.77–3.86	0.183	1.35	0.45–4.05	0.598
History of LAE				4.25	1.47–12.3	0.008
Non-missense variant	2.62	1.15–5.98	0.023	2.22	0.74–6.64	0.155

CI, confidence interval; ECG, electrocardiogram; HR, hazard ratio; LAE, lethal arrhythmia event; SND, sinus node dysfunction; SVT, supraventricular tachycardia.

**Figure 3:**
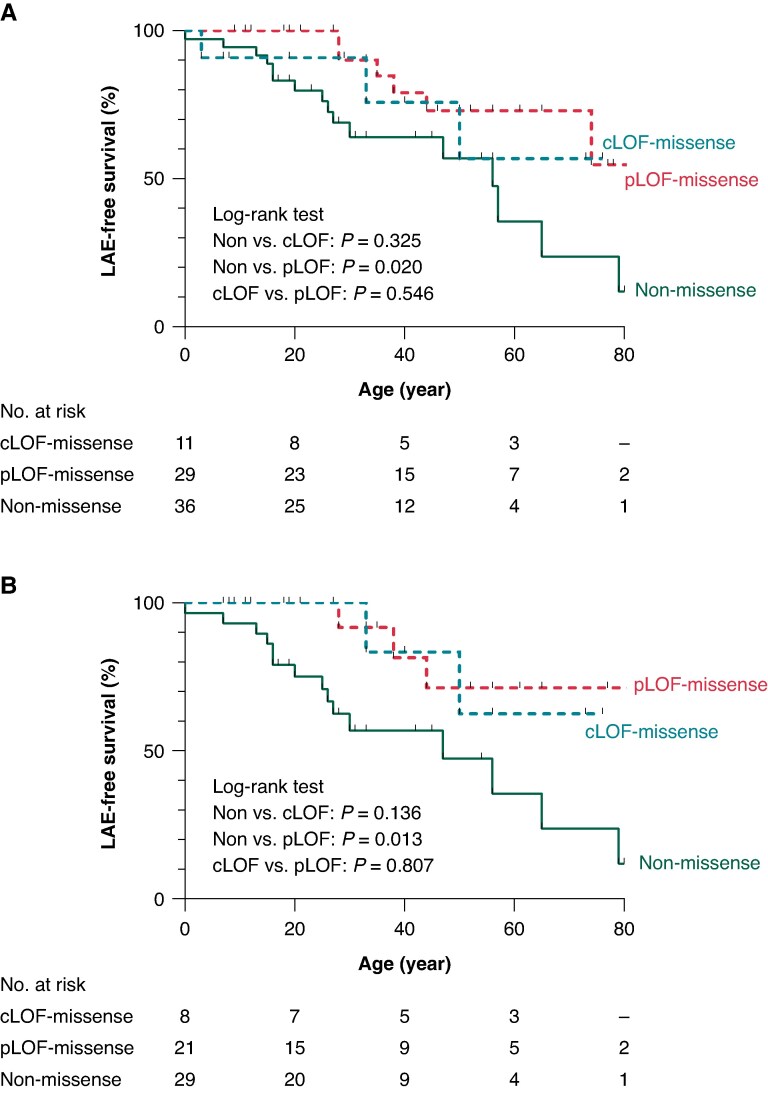
Kaplan–Meier survival analyses for the LAE-free survival. LAE-free survival estimates in lifetime for BrS patients with pLOF, cLOF and non-missense missense. Overall patients (*A*) and probands only (*B*). BrS, Brugada syndrome; cLOF, complete loss of function, LAE, lethal arrhythmia event; pLOF, partial loss of function.

To evaluate the effect of *I*_Na_ on prognosis, patients with missense variants were subdivided into those with cLOF-missense (*n* = 11) and pLOF-missense (*n* = 29) according to the amplitudes of *I*_Na_. In *Figure 3*, Kaplan–Meier analysis revealed that non-missense variants were linked to worse prognosis than pLOF-missense (log-rank *P* = 0.020). In contrast, when comparing them with cLOF-missense, there were no differences (log-rank *P* = 0.325). The same results were also observed in the analysis of probands (log-rank *P* = 0.013 and 0.136, respectively).

**Figure 4:**
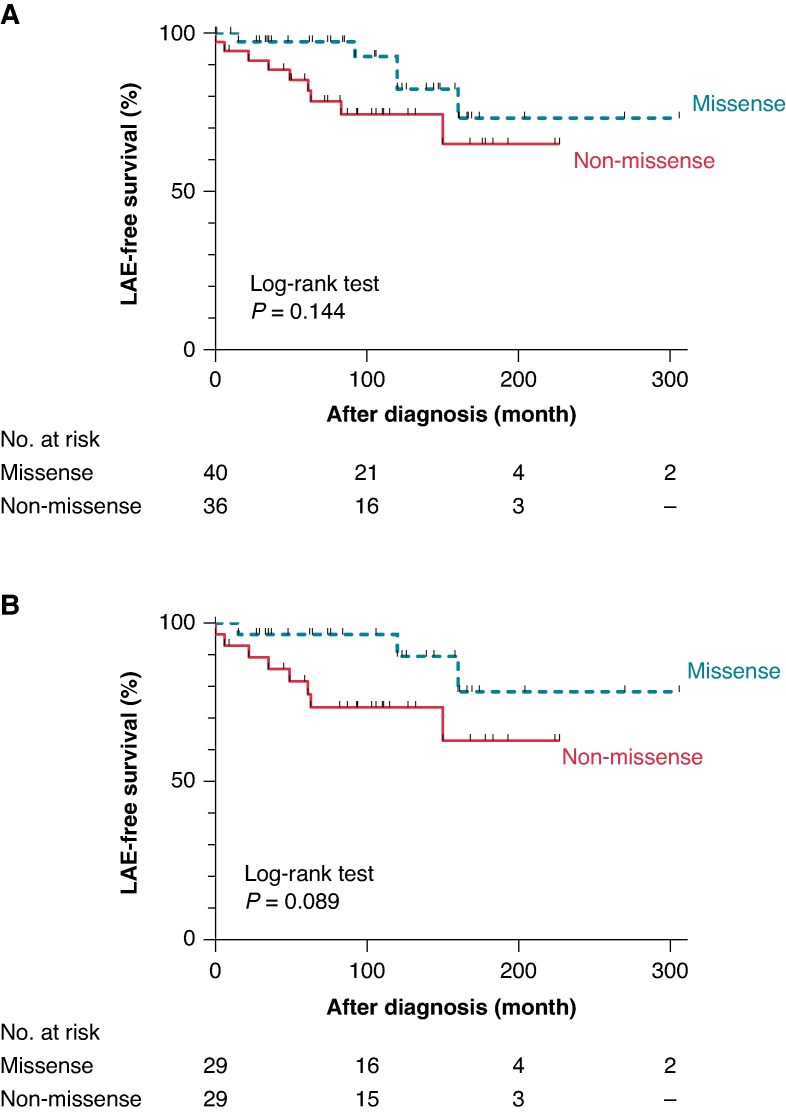
Kaplan–Meier survival analyses for the LAE-free survival after diagnosis. LAE-free survival estimates for BrS patients with missense and non-missense variants. Overall patients (*A*) and probands only (*B*). BrS, Brugada syndrome; LAE, lethal arrhythmia event.

*Figure 4* shows that after the detection of type 1 ECG in the patients, LAE-free survival rates between two groups were comparable in overall (log-rank *P* = 0.144) with an annual LAE rate of 1392/100 000 person-year and 2426/100 000 person-year for patients with missense and non-missense variants respectively, and analysis of only probands (log-rank *P* = 0.089). The univariate Cox regression (*Table 3*) analysis detected that only a history of LAE was a risk factor of LAEs after diagnosis (HR: 4.25, 95% CI: 1.47–12.3, *P* = 0.008), while the variant types did not affect the prognosis (HR: 2.22, 95% CI: 0.74–6.64, *P* = 0.155).

### Treatments

Implantable cardioverter defibrillators (ICDs) were implanted in 16 (21%) patients for primary prevention and 18 (24%) for secondary prevention. As shown in *Table 2*, patients with non-missense variants tend to undergo the ICD implantation for secondary prevention compared with those who harbour missense variants (33% vs. 15%, *P* = 0.061). Regarding pacemaker (PM) implantation, 11 patients were received PMs due to conduction disorders prior to diagnosis with BrS. Three of them upgraded to ICDs because of LAE experiences, and four of them did at battery replacement. Patients with non-missense variants received more PM implantation than those with missense variants (25% vs. 5%, *P* = 0.013). After the device implantations, 9 of 34 patients (26%) experienced device related complications: 6 inappropriate discharges due to SVTs, 2 device infections, and 4 lead-related problems.

At the final status, 17 (22%) patients started taking medication(s) and 11 (65%) continued to take medications. Only one patient underwent epicardial radiofrequency ablation therapy to control electrical storm, and after the operation, type 1 ECG pattern disappeared without recurrence of LAE to date.

## Discussion

### The impact of *SNC5A* non-missense variants on the clinical outcomes

*SCN5A* gene has been thought to be a sole causative gene associated with BrS phenotype,^7^ though it is identified in only 10–30% of BrS.^6^ Furthermore, a recent study that classified *SCN5A* variants based on functional analysis using patch-clamp methods revealed that the severity of functional impairment strongly correlated with prognosis.^10^ As the interpretation of the pathological significance of variants is refined, there are increasing reports of the prognostic value of *SCN5A* variants,^8,9^ including meta-analysis.^29,30^ However, it remains unevaluated whether different LOF-type *SCN5A* variants affect the clinical course of BrS patients.

In this study, based on the previous experimental results,^10^ we only selected ‘true’ LOF missense variants that generated reduced peak *I*_Na_ (<65% of the *I*_Na_ generated by WT) and evaluated if variant types can be a novel risk factor. In addition to relatively higher rate of overall LAEs (33%), our data showed that the BrS patients with non-missense variants showed poorer prognosis, such as LAEs and conduction disturbances, than those with missense variants. This result is associated with the *I*_Na_. Some missense variants can generate even a small amount of *I*_Na_, while most of non-missense variants are expected to produce no *I*_Na_. In fact, additional analyses showed that the prognosis of cLOF-missense carriers (*I*_Na_ = 0) was similar to that of non-missense carriers. *Figure 3* may indicate that the severity of *I*_Na_ dysfunction could be an important factor in determining patient outcomes.

Another explanation is that this finding might be attributed to the stoichiometry of the channel. Traditionally, Na_v_1.5 is believed to work as a monomer without interaction with other Na_v_1.5 channels. Therefore, the total amount of proteins may be an important determinant of the clinical features. However, non-missense variants exhibited several LOF mechanisms and some of them may escape NMD.^12,13^ Furthermore, it should be noted that differences in clinical prognosis based on the type of variants were not observed after patients were diagnosed despite the treatment including lifestyle implementations being the same, expecting it cannot contribute enough to improve scoring systems for predicting future LAEs.^28,31–33^ This may be caused by secondary factors beyond genetic influence, such as aging, sex hormones, or autonomic influences, and that’s why all patients with LOF-type *SCN5A* variants should be followed appropriately. As a number of studies have revealed that BrS has aspects of polygenic disease, single nucleotide polymorphism associated with BrS may also exert some prognostic impact.^34–39^

We also identified that non-missense variants are more likely to cause cardiac conduction disorder as shown in previous studies,^16–18^ resulting in higher rate of PM implantation. Several studies indicated that *SCN5A* showed a non-ionic function as well as an ionic function of Na_v_1.5 by being transported to not only T-tube but lateral cell membrane and intercalated disc, where they form complexes with other associated proteins and contribute mechanical connection of cardiomyocytes.^40–42^ Therefore, non-missense variants that change protein length and lose extensive connection sites may exhibit more frequent non-ionic dysfunction leading to additional structural changes similar to cardiac laminopathy,^43,44^ Marfan syndrome,^45^ and muscular dystrophy.^46^ Other studies showed that Na_v_1.5 in lateral membrane and intercalated disc generated greater *I*_Na_ than those in T-tubes, and the amount of *I*_Na_ still may be an important factor linked to abnormal conduction phenotypes.^42,47^ As BrS is considered to result from both repolarization and depolarization abnormalities,^37^ severe conduction disorder are likely to be directly linked to more severe clinical phenotypes.^16–18^

Our study population was relatively younger and was comprised of more female patients compared with the previously reported populations.^29,30,48^ This may be due to the inclusion of only LOF-type variants that can be more frequently detected in younger age and females.^49–51^ Regular ECG screenings of students in Japanese school help detect patients who harbour *SCN5A* variants.^52^

### The importance of follow-up of Brugada syndrome patients

During our follow-up periods of 9.0 [5.0–14.0] years, 18% patients who had been positive for pharmacological provocation tests showed spontaneous type 1 Brugada pattern ECG afterwards. Another study showed that 12% BrS patients with drug-induced type 1 ECG showed spontaneous ECG during 20-year follow-up.^53^ Furthermore, type 1 ECG appeared in seven patients with *SCN5A* variants, leading to new diagnosis with BrS and most of them (86%) had a family history of BrS. The penetrance of *SCN5A* positive BrS is not relatively low,^36,54^ however, we emphasize the importance of the regular check-up on BrS family members especially when they detected the same causative gene variants.

### Study limitations

We focused on previously reported *SCN5A* variants whose functions were studied, and therefore, the number of patients included in the analysis was relatively small. To determine the LOF variants, we mainly focused on the peak *I*_Na_ as a measure of functional damage. Therefore, we may have missed more complicated LOF of Na_v_1.5.^55–60^ Further studies with a larger number of patients, including their relatives harbouring variants, are necessary to provide convincing data.

## Conclusion

In this study, we showed, for the first time, that non-missense variants of the *SCN5A* were associated with poor prognosis than missense variants in BrS patients throughout lifetime, which may contribute to better clinical outcome measure based on variant types. Nevertheless, once patients were diagnosed with BrS, watchful follow-up was needed regardless of their variant types.

## Supplementary Material

euaf024_Supplementary_Data

## Data Availability

The data underlying this article will be shared on reasonable request to the corresponding author.
