# Correction to: *SCN5A* variant type-dependent risk prediction in Brugada syndrome

**DOI:** 10.1093/europace/euaf112

**Published:** 2025-06-05

This is a correction to: Takanori Aizawa, Takeru Makiyama, Hai Huang, Tomohiko Imamura, Megumi Fukuyama, Keiko Sonoda, Koichi Kato, Takashi Hisamatsu, Yuko Nakamura, Kenji Hoshino, Junichi Ozawa, Hiroshi Suzuki, Kazushi Yasuda, Hisaaki Aoki, Takashi Kurita, Yoko Yoshida, Tsugutoshi Suzuki, Yoshihide Nakamura, Yoshiharu Ogawa, Shintaro Yamagami, Hiroshi Morita, Shinsuke Yuasa, Masakazu Fukuda, Makoto Ono, Hidekazu Kondo, Naohiko Takahashi, Seiko Ohno, Yoshihisa Nakagawa, Koh Ono, Minoru Horie, *SCN5A* variant type-dependent risk prediction in Brugada syndrome, *EP Europace*, Volume 27, Issue 2, February 2025, euaf024, https://doi.org/10.1093/europace/euaf024

In the originally published version of this manuscript, an error was present in the graphical abstract.

The *P* value in the upper right graph of the graphical abstract was incorrectly given as *P* = 0.23, instead of *P* = 0.023.

This has now been corrected in the article.

