# Supplementary material for: SCN5A variant type-dependent risk prediction in Brugada syndrome
Source: Europace. 2025 Feb 11;27(2):euaf024. doi: 10.1093/europace/euaf024 (PMC11844247; doi:10.1093/europace/euaf024)
Supplement: euaf024_Supplementary_Data [file euaf024_supplementary_data.pdf]

Supplemental Materials

Supplemental Tables

Supplemental Table S1

References

Supplemental Tables

Supplemental Table S1 *SCN5A* LOF variants included in this study.

Codon change	Amino acid change	Domain	Variant type	peak I_{Na} %WT	MAF in gnomAD†	MAF in TogoVar‡	Number of families	Number of subjects	Reference
204T>A	Y68*		Nonsense	§	NA	NA	1	1	
481G>A	E161K	Transmembrane	Missense	23	4.15E-6	NA	2	2	1
535C>T	R179*		Nonsense	0	NA	NA	1	1	2
560C>T	T187I	Transmembrane	Missense	0	NA	NA	1	1	3
578G>A	W193*		Nonsense	§	NA	NA	1	1	
664C>T	R222*		Nonsense	0	NA	NA	1	1	4
845G>A	R282H	Pore	Missense	20	1.60E-5	NA	2	2	3
844C>T	R282C	Pore	Missense	1	NA	NA	1	1	3
870delC	T290fs*53		Frameshift	0	NA	NA	1	2	5
1066G>A	D356N	Pore	Missense	1	4.02E-6	NA	2	2	3
1099C>A	R367S	Pore	Missense	0	NA	NA	1	1	6
1330_1331del	E444fs*14		Frameshift	§	NA	NA	1	1	
1338G>A	E446Espl		Splicing error	§	NA	NA	1	1	¶
1603C>T	R535*		Nonsense	§	NA	NA	1	2	
2271delA	I759Ffs*5		Frameshift	§	NA	NA	1	1	
2335C>T	Q779*		Nonsense	§	NA	NA	1	1	
2531delT	L844Rfs*3		Frameshift	§	NA	NA	1	1	
2677C>T	R893C	Pore	Missense	8	1.06E-5	NA	2	4	3
2678G>A	R893H	Pore	Missense	0	3.99E-6	NA	1	1	7

2711G>A	W904*		Nonsense	§	NA	NA	1	2	
2729C>T	S910L	Pore	Missense	1	3.99E-6	NA	2	4	3
3284G>A	W1095*		Nonsense	§	NA	NA	1	1	
3673G>A	E1225K	Transmembrane	Missense	41	4.01E-6	NA	2	2	3
3743_3744del	Y1248Cfs*69		Frameshift	§	NA	NA	1	2	
4012_4014del	L1338del	Pore	In-frame	0	NA	NA	1	2	¶
4035G>T	W1345C	Pore	Missense	12	NA	NA	1	2	8
4037_4038del	L1346Hfs*38		Frameshift	§	NA	NA	1	1	
4186A>C	T1396P	Pore	Missense	0	NA	NA	1	1	¶
4222G>A	G1408R	Pore	Missense	0	NA	NA	1	1	9
4258G>C	G1420R	Pore	Missense	3	NA	NA	1	3	3
4346A>G	Y1449C	Pore	Missense	10	NA	NA	1	1	8
4534C>T	R1512W	Interdomain	Missense	69, 50[§§]	5.57E-5	NA	1	1	10
4720G>A	E1574K	Transmembrane	Missense	39	NA	NA	1	2	3
4732_33dup	K1578fs*52		Frameshift	0	NA	NA	1	1	11
4867C>T	R1623*		Nonsense	0	NA	NA	2	2	11
4912C>T	R1638*		Nonsense	0	7.95E-6	NA	1	1	12
4972_4975dup	N1659Ifs*131		Frameshift	§	NA	NA	1	1	
5218G>A	G1740R	Pore	Missense	30	NA	NA	1	1	3
5227G>A	G1743R	Pore	Missense	0	NA	NA	3	6	9
5321_5324dup	F1775Lfs*15		Frameshift	§	NA	NA	2	3	
5350G>A	E1784K	C-terminus	Missense	60	NA	NA	1	1	9

5423_5424insA	F1808Lfs*3		Frameshift	§	NA	NA	1	1	
5575A>G	K1859E	C-terminus	Missense	60	NA	NA	1	1	¶
del exon 1-28			CNV	§	NA	NA	1	2	
del exon 4			CNV	0	NA	NA	1	1	1
del exon 7-12			CNV	§	NA	NA	1	1	
dup exon 17-24			CNV	§	NA	NA	1	1	
del exon 24			CNV	0	NA	NA	1	1	13

†We described the total allele count of all the ethnic groups in gnomAD v2.1.1.

‡We described the summed-up numbers of the Japanese datasets (GEM Japan Whole Genome Aggregation and Human Genetic Variation Database) in TogoVar databases.

§Sodium current reduction is predicted to be 100% (based on type of mutation, i.e., truncation and/or frameshift between exon 2 to 27)

§§This variant decreased peak I_{Na} by around 50% compared to WT under the acidosis condition.

¶This variant was studied in our Laboratory (unpublished data, data was not shown)

CNV depicts copy number variation; cLOF, complete LOF; LOF, loss of function; MAF, minor allele frequency; WT, wild type.

References

1. Sonoda K, Ohno S, Ozawa J, Hayano M, Hattori T, Kobori A, *et al*. Copy number variations of SCN5A in Brugada syndrome. *Heart Rhythm* 2018; 15:1179-1188.
2. Kawamura M, Ozawa T, Yao T, Ashihara T, Sugimoto Y, Yagi T, Itoh H, Ito M, Makiyama T, Horie M. Dynamic change in ST-segment and spontaneous occurrence of ventricular fibrillation in Brugada syndrome with a novel nonsense mutation in the SCN5A gene during long-term follow-up. *Circ J* 2009; 73:584-8.
3. O'Neill MJ, Muhammad A, Li B, Wada Y, Hall L, Solus JF, *et al*. Dominant negative effects of SCN5A missense variants. *Genet Med* 2022; 24:1238-1248.
4. Ortiz-Bonnin B, Rinné S, Moss R, Streit AK, Scharf M, Richter K, Stöber A, Pfeufer A, Seemann G, Kääb S, Beckmann BM, Decher N. Electrophysiological characterization of a large set of novel variants in the SCN5A-gene: identification of novel LQTS3 and BrS mutations. *Pflugers Arch* 2016; 468:1375-87.
5. Kato K, Makiyama T, Wu J, Ding WG, Kimura H, Naiki N, Ohno S, Itoh H, Nakanishi T, Matsuura H, Horie M. Cardiac channelopathies associated with infantile fatal ventricular arrhythmias: from the cradle to the bench. *J Cardiovasc Electrophysiol* 2014; 25:66-73.
6. Suzuki K, Sonoda K, Aoki H, Nakamura Y, Watanabe S, Yoshida Y, *et al*. Association Between Deleterious SCN5A Variants and Ventricular Septal Defect in Young Patients With Brugada Syndrome. *JACC Clin Electrophysiol* 2022; 8:297-305.
7. Kapplinger JD, Giudicessi JR, Ye D, Tester DJ, Callis TE, Valdivia CR, *et al*. Enhanced Classification of Brugada Syndrome-Associated and Long-QT Syndrome-Associated Genetic Variants in the SCN5A-Encoded Na(v)1.5 Cardiac Sodium Channel. *Circ Cardiovasc Genet* 2015; 8:582-95.
8. Glazer AM, Wada Y, Li B, Muhammad A, Kalash OR, O'Neill MJ, *et al*. High-Throughput Reclassification of SCN5A Variants. *Am J Hum Genet* 2020; 107:111-123.
9. Ishikawa T, Kimoto H, Mishima H, Yamagata K, Ogata S, Aizawa Y, *et al*. Functionally validated SCN5A variants allow interpretation of pathogenicity and prediction of lethal events in Brugada syndrome. *Eur Heart J* 2021; 42:2854-2863.
10. Zheng J, Zhou F, Su T, Huang L, Wu Y, Yin K, *et al*. The biophysical characterization of the first SCN5A mutation R1512W identified in Chinese sudden unexplained nocturnal death syndrome. *Medicine (Baltimore)* 2016; 95: e3836.
11. Makiyama T, Akao M, Tsuji K, Doi T, Ohno S, Takenaka K, Kobori A, Ninomiya T, Yoshida H, Takano M, Makita N, Yanagisawa F, Higashi Y, Takeyama Y, Kita

T, Horie M. High risk for bradyarrhythmic complications in patients with Brugada syndrome caused by SCN5A gene mutations. *J Am Coll Cardiol* 2005; 46:2100-6.

12. Kosmidis G, Veerman CC, Casini S, Verkerk AO, van de Pas S, Bellin M, Wilde AA, Mummery CL, Bezzina CR. Readthrough-Promoting Drugs Gentamicin and PTC124 Fail to Rescue Nav1.5 Function of Human-Induced Pluripotent Stem Cell-Derived Cardiomyocytes Carrying Nonsense Mutations in the Sodium Channel Gene SCN5A. *Circ Arrhythm Electrophysiol* 2016; 9: e004227.
13. Schroeter A, Walzik S, Blechschmidt S, Haufe V, Benndorf K, Zimmer T. Structure and function of splice variants of the cardiac voltage-gated sodium channel Na(v)1.5. *J Mol Cell Cardiol* 2010; 49:16-24.
